# Safety and efficacy of INTRABEAM intraoperative radiotherapy for invasive thymoma

**DOI:** 10.1097/MD.0000000000020964

**Published:** 2020-07-02

**Authors:** Tian-xiang Cui, Ji-gang Dai, Jing-meng Li, Jin-dong Qian, Guang-hui Li, Jian-guo Sun

**Affiliations:** a Cancer Institute of PLA; b Department of Thoracic Surgery, Xinqiao Hospital, Army Medical University, Chongqing, China.

**Keywords:** INTRABEAM, intraoperative radiotherapy, surgery, thymoma

## Abstract

Intraoperative radiotherapy (IORT) has been used to treat different residual solid tumors after tumor removal and has shown many advantages over other treatment methods. However, the use of IORT for invasive thymoma has not been reported. Therefore, in this study, we tried to determine the safety and efficacy of INTRABEAM IORT for the treatment of invasive thymoma.

Among the patients admitted to our hospital from September to December 2016 who were diagnosed with invasive thymoma, 14 were selected as study subjects. With medical histories taken beforehand, 8 of these patients were diagnosed with Masaoka stage IIA and 6 with Masaoka stage IIB; furthermore, 5 of the patients were diagnosed with myasthenia gravis (MG). INTRABEAM radiation (8–10 Gy, low energy) was delivered to the postoperative tumor bed of each patient during surgery. The intra- and postoperative complications were observed and evaluated, and the improvement in symptoms was assessed. An additional 23 patients with stage II thymoma undergoing radical surgery from April to August 2016 were chosen as the control group.

One month after the operation, only 1 patient in the IORT group had cough, increased levels of leucocytes and neutrophils, and pulmonary inflammation on chest computed tomography. Reactive inflammation and pleural effusion in the 2 groups were similar (*P* > .05). There was no significant difference between the 2 groups in the improvement of myasthenia gravis (*P* > .05). Postoperative chest computed tomography and routine blood examination at 3 and 12 months showed that all the patients recovered, with normal hemogram levels and no pulmonary fibrosis around the radiation field. In addition, ultrasonic cardiography and electrocardiography demonstrated no significant difference before or after surgery within the IORT group. At the end of the follow-up, all the patients were alive, no relapse or remote metastasis was observed in the IORT group, and 2 inpatients in the control group had experienced relapse at 24 and 26 months. There was a significant difference in disease-free survival between the 2 groups (*P* = .00).

It is safe to administer low-energy INTRABEAM IORT at a dose of approximately 10 Gy in patients with stage II invasive thymoma. INTRABEAM IORT does not significantly increase operation- or radiation-related complications and has no significant effect on vital organs such as the lungs and heart. Its long-term efficacy is worth expecting.

## 1 Introduction

Intraoperative radiotherapy (IORT) is the application of a single dose of radiation to the tumor bed, possibly the affected site, and the lymphatic drainage area that are exposed during surgery in a single session.^[1]^ A large dose in a single session can generate 2 to 3 times more biological effects than fractionated external beam radiation therapy (EBRT).^[2]^ For example, a single dose of IORT with 20 Gy is biologically equivalent to a 70-Gy dose of EBRT.^[3]^ Moreover, with collaboration of surgeons and radiation therapists, IORT can be delivered immediately after resection of tumor tissue and directly to the target region, thus shortening the interval between surgery and radiation and decreasing the proliferation of tumor cells. Furthermore, the radiation field can be precisely determined by moving the normal tissues out of the field of application or covering them, thus greatly reducing the damage to surrounding tissues and the incidence of radiation-related complications.^[4–6]^

Given the above benefits, IORT has been widely applied in the treatment of breast cancer, pancreatic cancer, gastric cancer, rectal cancer, and solitary brain metastasis in recent years and has demonstrated its superiority to other post-tumor removal treatments.^[7–11]^ However, to the best of our knowledge, no application of IORT for invasive thymoma has been reported.

Therefore, we recruited 14 patients from all cases of stage II thymoma identified by pathological examination admitted to our hospital from September 1 to December 31, 2016. The patients were administered INTRABEAM IORT (8–12 Gy, median dose 10 Gy, 50 KVe) to the tumor bed after radical surgery for thymoma. The safety and efficacy of IORT were investigated.

## 2 Materials and methods

### 2.1 Patient collection

Patients were collected from September to December 2016. For preliminary selection, the patients who consulted the Department of Thoracic Surgery in our hospital due to a mediastinal mass and myasthenia gravis (MG) were considered candidate subjects of the current study. With a mediastinal mass confirmed by thoracic CT scanning and remote metastasis ruled out, these candidates were assigned to receive surgical treatment. For secondary selection, patients included in the study met the following criteria:

1. intraoperative frozen section-confirmed thymoma;
2. capsular invasion or visible organ lesion; and
3. Masaoka stage II thymoma.

The pathological subtype and Masaoka stage of the tumor were determined by postoperative pathological examination. Through the end of December 2016, 14 patients were enrolled and underwent IORT. For a historical comparison, we also chose recent cases with the same enrollment criteria who had not received IORT from April to August 2016 as the control group. Finally, 23 control patients were included. Each patient signed an informed consent form, and all procedures were conducted with approval from the Ethics Committee of Xinqiao Hospital, Army Medical University. This study was performed in accordance with the Declaration of Helsinki, as revised in 2013.

### 2.2 Surgical procedures

All surgical procedures were completed under a video-assisted thoracoscope.^[12]^ Patients were treated with a right thoracic approach and placed in a left lateral recumbent position at 30 to 45 degrees. The operation was completed through 2 ports in the chest wall. Surgical margins were negative in all cases, up to the criteria for R0. In the IORT group, a 1.5 to 2cm port for observation and auxiliary operation was created between the 5th or 6th intercostal space along the right midaxillary line, and a 2 to 4cm port for major operation was created in the 3rd or 4th intercostal space along the right anterior axillary line. An incision protection retractor was attached to the major operation port. After insertion of the instruments, extended thymectomy was performed.

### 2.3 INTRABEAM IORT technique

**Figure 1:**
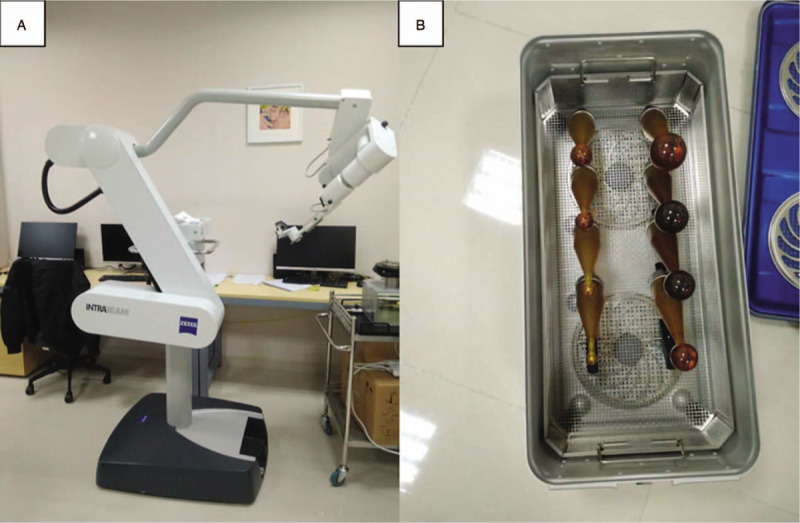
INTRABEAM device (A) and spherical applicators with cone-shaped shanks (B).

IORT in the current study was administered by using INTRABEAM. With a flexible arm and a balanced stand, the INTRABEAM treatment system (Carl Zeiss, Oberkochen, Germany, Fig. 1A & B) is a mobile miniature electron beam-driven device designed to deliver radiation directly to the tumor bed during surgery.^[13]^ It can provide a point source of low energy X-rays (50 kV max) at the tip of an electron beam in a drift tube (3.2 mm in diameter) fixed at the end of the manipulator. The tube is sheathed by a spherical applicator (ranging from 1.5 to 5.0 cm in diameter) with a cone-shaped shank at the bottom, which is placed upside-down during IORT.^[14,15]^ Low-energy X-rays are emitted after the electron beam hits the gold target at the tip of the tube and are modulated by the spherical applicator to deliver a uniform dose.^[14]^

**Figure 2:**
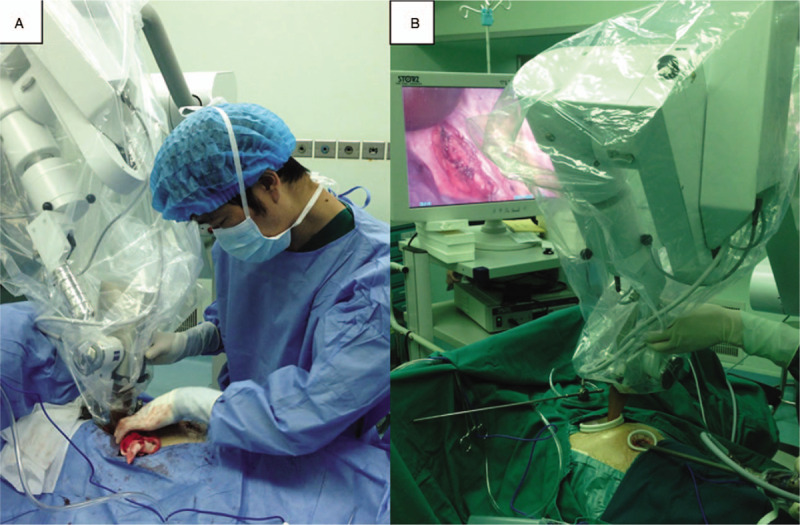
The surgeon placed an applicator of proper size above and against the tumor bed through the major operation port (A and B).

Based on the specific size of the tumor and tumor bed, an applicator of appropriate size was deployed above and against the tumor bed. A saline-soaked gauze was then folded into a square of 2 cm thickness and placed around the applicator to prevent the normal tissues from the X-rays, thus ensuring the localization of radiation to the tumor bed area (Fig. 2A & B).

In total, a single dose of 8 to 10 Gy at the applicator surface was administered in the IORT group. The specific radiation time was determined by comprehensively considering the radiation dose, depth of invasion and size of the applicator. The different IORT parameters are shown in Table 1.

**Table 1:**
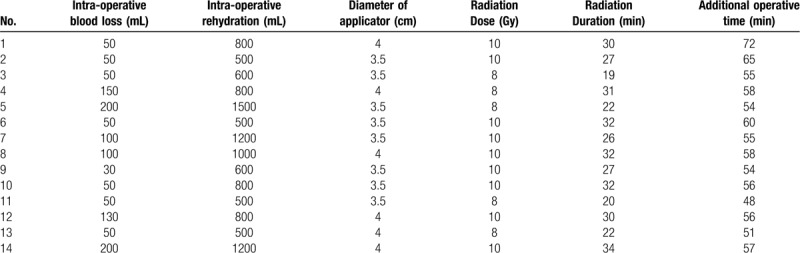
Surgical conditions of patients and intraoperative radiotherapy parameters of the Intraoperative radiotherapy group.

### 2.4 Follow-up and assessment

All 14 patients in the IORT group were required to return for examination 1 month, 3 months, and 1 year after discharge from the hospital. The examination included routine blood tests, liver and kidney function tests, computed tomography (CT) scanning of the lungs, electrocardiography, ultrasonic cardiography, etc. The 23 patients in the control group underwent lung CT scans 1 month after discharge from the hospital. After 1 year, all patients were followed up every 3 months by phone call or outpatient consultation. The requested content included improvements in the myasthenia gravis, survival, quality of life, etc.

### 2.5 Statistical analysis

All statistical analyses were performed using SPSS, version 20.0 (SPSS, Inc., Chicago, IL). The median follow-up was calculated with the Kaplan–Meier method. Differences were considered to be statistically significant at *P* < .05.

## 3 Results

### 3.1 General characteristics of patients

IORT group: 14 patients were observed in the current study, consisting of 9 males and 5 females, with a median age of 53 years (range: 31–72). According to the Masaoka staging system, 8 patients had stage IIA thymoma, and 6 had stage IIB thymoma. According to pathological type, 2 patients had B1 type, 6 had B2 type, and 6 had AB type. Five patients had comorbid myasthenia gravis (MG). The detailed data on the general characteristics are listed in Table 2.

**Table 2:**
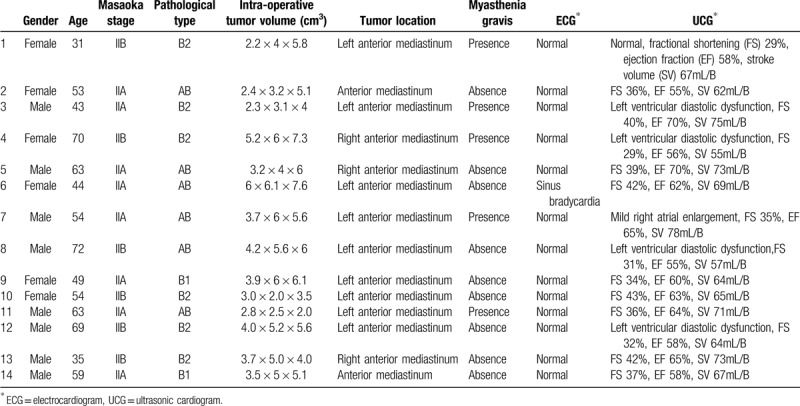
General characteristics of the intraoperative radiotherapy group patients.

Control group: The control group consisted of 14 males and 9 females with a median age of 47 years (range: 22–72). According to the Masaoka staging system, 15 patients had stage IIA thymoma, and 8 had stage IIB thymoma. According to pathological type, 4 patients had B1 type, 8 had B2 type, 10 had AB type, and one patient was untyped. Seven patients had comorbid MG.

### 3.2 Short-term complications

IORT group: Blood volume loss and rehydration during the surgery are shown in Table 1. The times for installation and operation of the radiation equipment are listed in Table 1, with the maximum time being 72 minute, the minimum 48 minute, and the mean 57.6 minute. No serious complications associated with surgery or IORT were observed in the 14 patients, and no postoperative myasthenic crisis was noted in the 5 patients with myasthenia gravis.

Control group: No serious complications associated with surgery were observed in the 23 patients, and no postoperative myasthenic crisis was noted in the 7 patients with MG.

### 3.3 Long-term complications

**Figure 3:**
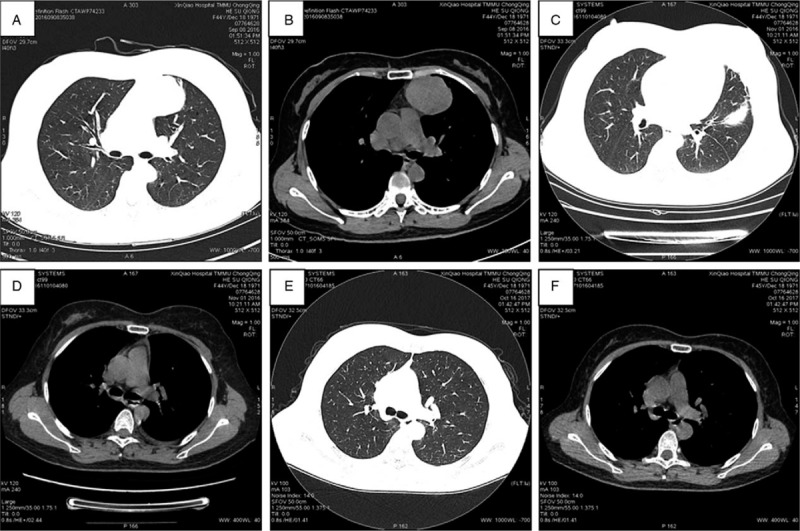
Preoperative chest computed tomography (CT) shows a mass in the left anterior mediastinum (A and B); chest CT at postoperative month 1 shows pulmonary infection in the left upper lobe (C) and a small amount of pleural fluid on the left side (D); and chest CT at postoperative month 6 shows normal lungs and no pulmonary fibrosis in the irradiated region (E and F).

IORT group: During the follow-up, CT scanning of the lungs 1 month after discharge revealed that 5 patients had developed moderate pulmonary inflammation, of whom 1 had cough and increased levels of leucocytes and neutrophils, and 2 had a small amount of pleural fluid. However, CT scanning of the lungs and routine blood examination 3 and 12 months after discharge showed that all the patients had returned to normal, without increased hemogram levels, cough, shortness of breath on exertion, or pulmonary fibrosis around the radiation field. For example, in patient No. 6, a mass was shown in the left anterior mediastinum before surgery (Fig. 3A & B). One month after surgery and IORT, pneumonia in the left upper lobe and a small amount of pleural effusion on the left side were found (Fig. 3C & D). After antibiotic treatment and on chest CT 6 months later, there was no pneumonia or pulmonary fibrosis (Fig. 3E & F). In addition, ultrasonic cardiography and electrocardiography demonstrated no significant difference before and after surgery in the IORT cohort (Table 3).

**Table 3:**
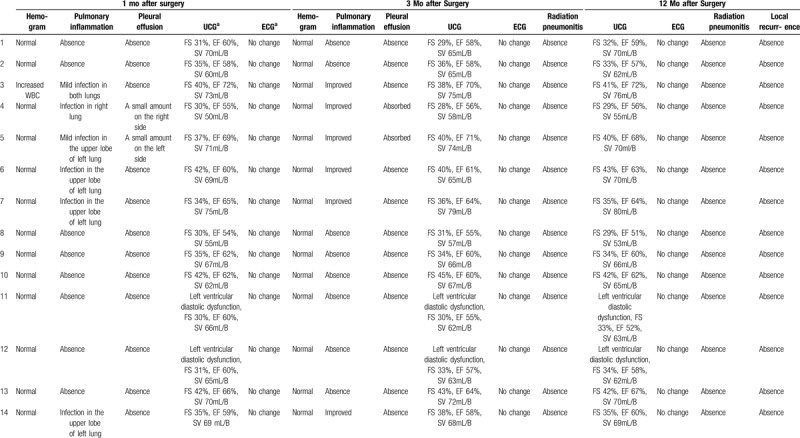
Conditions of each patient during the follow-up in the intraoperative radiotherapy group.

Control group: 7 patients developed moderate pulmonary inflammation, and 3 had a small amount of pleural fluid on CT scanning of the lungs 1 month after discharge. Reactive inflammation and pleural effusion in the 2 groups were similar (*P* > .05, Table 4).

**Table 4:**
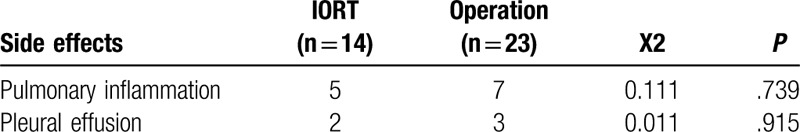
Side effects in the 2 groups.

### 3.4 Outcomes

**Figure 4:**
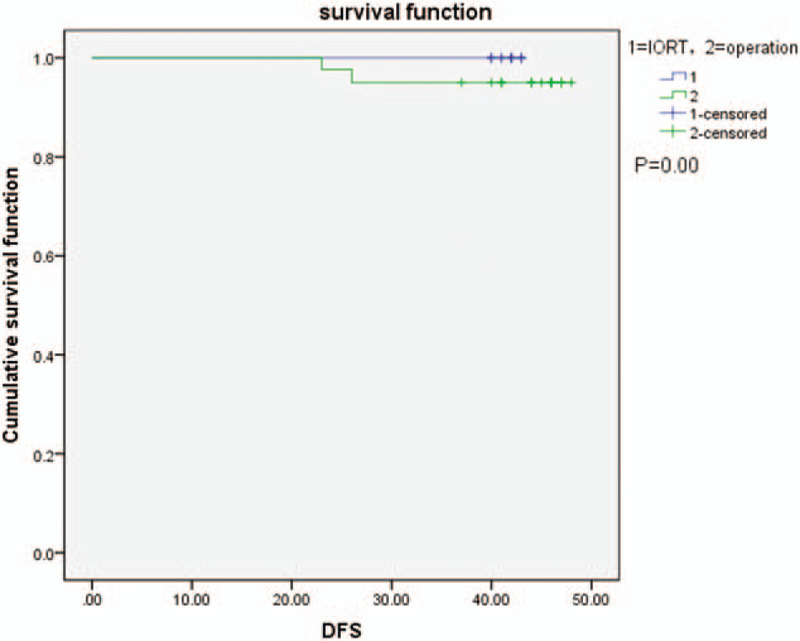
Disease-free survival curves of the 2 groups. There was a significant difference in the disease-free survival between the 2 groups (*P* = .00).

The last follow-up date was April 30, 2020, with a whole process of 40 to 43 months and a median duration of 41 months in the IORT group and a whole process of 41 to 47 months and a median duration of 45 months in the control group. The IORT group demonstrated no relapse or remote metastasis throughout the follow-up. The control group had 2 patients with relapse at 24 and 26 months. The median disease-free survival (DFS) was not reached in either group by the last follow-up. There was a significant difference in the DFS curves between the 2 groups (*P* < .001, Fig. 4). Among the 5 patients with myasthenia gravis in the IORT group, 4 significantly improved after treatment. Only 1 patient did not show obvious improvement in symptoms and was given oral neostigmine to control blepharoptosis and the inability to comb hair. The control group had 7 patients with MG, all of whom significantly improved after treatment. There was no significant difference between the 2 groups in the improvement of myasthenia gravis (*P* = .217).

## 4 Discussion

Thymoma is derived from the thymic epithelium and accounts for approximately 50% of all types of anterior mediastinal tumors. Although most thymomas are benign, they have the potential to transform into malignancies that can invade local tissue or metastasize to remote sites. According to statistics from the National Cancer Institute, the incidence of thymoma is 0.13 per 100,000 person-years in the United States.^[16]^ In addition, over 30% of patients with thymoma have a comorbidity of myasthenia gravis.^[17]^ Consistent with the previous report, myasthenia gravis (MG) was present in 5 of 14 in the IORT group and 7 of 23 in the control group in the current study. Therefore, it is vitally important to treat thymoma properly.

Surgical removal is the standard treatment for thymomas that can be completely excised. However, whether the tumor can be completely removed remains a problem and determines the prognosis of the patients. It has been reported that the completeness of surgical resection is a factor that substantially affects local recurrence and survival.^[18]^ According to the International Thymic Malignancy Interest Group, complete resection can only be achieved by removing the whole thymus and surrounding tissue.^[19]^

To enhance the effects of comprehensive treatment, other adjuvant therapies are also used, such as postoperative radiotherapy (PORT).^[20]^ The National Comprehensive Cancer Network 2018 guidelines for thymoma recommend that the use of postoperative adjuvant radiation for reducing local recurrence after R0 resection of stage II to IV thymoma. However, the role of PORT remains unclear for the complete resection of stage II thymoma. Some studies have reported that PORT could significantly improve the prognosis of patients with stage II or III thymoma,^[21]^ whereas it is not helpful in decreasing the local recurrence or increasing the survival of patients with stage II or even more invasive thymoma.^[22]^ Consistent with those results, after analyzing 1320 patients with stage II or III thymoma, Kondo et al found that PORT neither significantly inhibited local recurrence nor improved patient outcome.^[23]^

Once the thymoma breaks through its envelope or invades the mediastinal adipose tissue at stage II, the rate of local recurrence and metastasis rise to 11%.^[24]^ Consistent with the previous report, in our study, the control group included 2 patients who had a relapse at 24 and 26 months. Compared with the control group, the IORT group patients with stage II thymoma showed improved DFS. Therefore, radiation therapy is still needed in combination with surgical removal, but the time of delivery can be adjusted.

Since IORT is more precise and used earlier than PORT, it can be considered to optimize the treatment. Simultaneously with surgery, radiation can be precisely and timely delivered to the tumor bed and surrounding tissue, thus maximally protecting the normal tissue and minimizing the time for tumor proliferation.^[25]^ Furthermore, IORT can inhibit local recurrence by changing the tumor microenvironment. Exudates collected at surgical wounds within 24 hours after breast-conserving surgery were shown to stimulate the proliferation and migration of breast cancer cells in vitro, while those from wounds that underwent IORT could not.^[25]^ Although the single dose of IORT is much higher than that of EBRT, low-energy X-rays have weaker penetrating power; when the radiation dose for the tumor bed was 20 Gy, the power would decrease to 5 to 7 Gy 1 cm from the surface.^[13]^ Deployment of a spherical radiation applicator at a certain distance from the skin and the thoracic wall can effectively reduce radiation injury, thus making radiation protection easier than in EBRT. In the current study, we protected normal tissues by covering the radiation applicator with a wet gauze of 2 cm thickness.

After the surgery, we closely followed up the patients and found that it is safe to deliver INTRABEAM IORT to the tumor bed at a dose of approximately 10 Gy with low-energy X rays. The extension of the operation time did not significantly increase radiation-related complications. Although 5 patients presented with pulmonary infections after surgery, only 1 of them had cough and increased levels of leukocytes and neutrophils, which met the diagnostic criteria of pulmonary infection, and the other 4 were most likely due to inflammatory responses after surgery. Until the end of the follow-up on Apr 30, 2020, no radiation pneumonitis or pulmonary fibrosis around the radiation field and no tumor recurrence had been found in any patient.

Moreover, because thymoma is located in specific places, generally near the heart and great vessels, intraoperative radiation may exert effects on the conduction system and the systolic and diastolic functions of the heart. We therefore compared the ultrasonic cardiograms before IORT with those after IORT at 6 months and 1 year. No significant change was observed in the systolic and diastolic functions or chamber size of the heart, suggesting that IORT does not affect cardiac function. Similarly, no obvious change was detected in the electrocardiogram before and after surgery, indicating that IORT does not affect the cardiac conduction system.

In summary, the current study has demonstrated the safety of applying INTRABEAM IORT in patients with invasive thymoma. The aim of this study was to explore the safety and primary efficacy; thus, we did not have a large sample size in this study. Due to the limited sample size and the non-randomized controlled trial design of the study, we plan to design a prospective trial to confirm the effectiveness of INTRABEAM IORT in treating invasive thymoma in the near future.

## Acknowledgments

We thank The National Key Research and Development Project, 2016YFC0106400; Clinical research Foundation of Army Medical University, 2018XLC1010.

## Author contributions

**Administrative support:** Jian-Guo Sun and Guang-hui Li.

**Collection and assembly of data:** Jin-dong Qian.

**Conception and design:** Tian-xiang Cui.

**Data analysis and interpretation:** Tian-xiang Cui.

**Provision of study materials or patients:** Jing-meng Li and Ji-gang Dai.
